# Supplementary material for: Heart Rehabilitation for All (HeRTA): Protocol for a feasibility study and pilot randomized trial
Source: PLoS One. 2022 Jun 17;17(6):e0270159. doi: 10.1371/journal.pone.0270159 (PMC9205521; doi:10.1371/journal.pone.0270159)
Supplement: S1 Protocol — (DOCX) [file pone.0270159.s002.docx]

# Heart Rehabilitation for All (HeRTA)

## A feasibility study

## Introduction

HeRTA is a partnership project conducted in close collaboration between Hvidovre Hospital, Rehabilitation Center Albertslund (municipality), local sports organizations, the Danish Heart Association, and Center for Clinical Research and Prevention (CCRP). A patient advisory board is involved throughout the project to ensure patient involvement including that decisions continuously focus on patient needs.

**The overall aim of HeRTA** is to develop and test the feasibility of a new, sustainable model for rehabilitation supporting vulnerable patients to take part in rehabilitation and promoting life-long activity for all patients with heart disease.

More specifically our goal is to:

- test if combined activities across sectors can increase the proportion of cardiac patients’ participating in rehabilitation
- test if the model improves the maintenance of lifestyle changes and enhances physical and mental functioning, quality of life, and self-care capacity among cardiac patients

The project is organized in three phases:

1. **An ongoing development phase**, where all partners including the patient advisory board participate in a partnership and co-creation process to develop model content and collaboration procedures
2. **A feasibility phase** with a small scale RCT-component, where we examine whether the intervention activities are feasible, acceptable, and may have positive effects for patients with heart disease
3. **A long-term follow-up and implementation phase**, in which sustainability of the intervention on patient activity is assessed and promising components are further tested.

## 2. Background

Half a million Danes suffer from heart diseases^1^, and heart disease often coincides with other chronic conditions, with the combination of heart disease and diabetes being the most prominent^2^. According to national guidelines, people with heart diseases should be offered rehabilitation to minimize consequences and prevent new cardiac episodes. Solid evidence exists on the benefits of multifaceted cardiac rehabilitation on patients' cardiovascular function, functional level, and survival^3^.

Today, half of the cardiac patients do not participate in rehabilitation, and vulnerable patients are more likely to decline participation^4^. Structural barriers during the transition between hospital and municipality make it difficult for patients to navigate in rehabilitation activities. Additionally, the probability of being offered rehabilitation is lower if patients live alone, are unemployed, have a short education, a low income or suffer from several chronic conditions^4^. Other barriers to participation are a poor financial situation, weak social relationships, logistic challenges, language difficulties, and cultural considerations^4–7^. Even after having participated in rehabilitation programs, many patients struggle to maintain new lifestyle habits^8^.

Currently, the content of cardiac rehabilitation is disease management, medical treatment, tobacco cessation support, physical exercise, psychosocial support, and guidance about alcohol and nutrition. Despite health professionals’ intentions to address the vulnerability, vulnerable patients are overrepresented among those who do not receive a referral to rehabilitation, who do not participate in rehabilitation, and who do not complete rehabilitation activities.

## 3. Feasibility phase

During the development phase, partners and a patient advisory board has developed a model of combined activities aimed at creating a coherent rehabilitation course addressing needs among all patients with a special focus on patients with vulnerability. The model has used identified barriers for rehabilitation participation and benefits as a starting point.

The rehabilitation process is initiated 14 days after discharge with rehabilitation needs assessment. At this point, all patients will receive an *information book* that provides relevant and thorough information and contact information to patients. The information book also contains information on the availability of *patient supporters* from the Heart Association that provide a 1:1 conversation to patients who wishes to talk to an experienced patient about their worries concerning life with cardiac disease. *Employer material* will help patients inform employers and gain their support for rehabilitation participation and a plan for returning to work. *Support café for relatives* will support relatives in dealing with their worries as well as in supporting cardiac patients.

Patients with vulnerabilities will have access to *patient education conducted in small groups* that utilize patient involving methods to target and provide information relevant to patients’ daily lives. In addition, patients with vulnerabilities may be referred to *pro-active counselling* where a cardiac nurse, psychologist, or social worker from the Heart Association calls the patient to provide targeted support to their specific worries and challenges. Finally, patients with vulnerabilities may receive *paid transportation* to ensure that finances are not an obstacle for commuting to Rehabilitation Center Albertslund.

During rehabilitation at hospital and municipality, collaboration with civil society provides a safe *transition to local sports associations* and the Heart Association’s *local area exercise groups*. *Follow up phone calls* from physiotherapists in the municipality support patients to continue physical activity.

### Design, materials, and methods

We use qualitative data on implementation and acceptability of intervention among partners and patients. An RCT component will assess potential effects on patient participation rates, health, and life quality. The process evaluation will be guided by Normalization Process Theory (NPT)^9^ and the Consolidated Framework for Implementation Research (CFIR)^10^. To ensure successful implementation and sustainability, partners will meet quarterly to evaluate experiences with patient pathways and collaboration across partners. At these meetings, necessary adjustments are agreed upon. In the initial three months, meetings are more frequent to allow for relevant adjustments. In the remaining 9 months, strong rationale for changes should be provided to protect the potential of the study’s RCT component. All changes in activities or procedures throughout the feasibility phase - including the rationale for adjustments - will be registered in field notes.

##### Patient recruitment

Patients, who meet the inclusion criteria:

1. Ischaemic heart disease, cardiac valve surgery, persistent atrial fibrillation, or heart failure
2. Resident in Hvidovre Hospitals uptake area
3. Cognitively functional
4. Physically able to participate in rehabilitation activities

will be invited to participate in HeRTA by health care professionals at the cardiac department, Hvidovre Hospital. If they accept, they will receive a link to REDCap^12^ where they are asked to consent to a) information exchange between partners and b) participation in the project. Vulnerability is assessed at baseline and defined as either a) a cut-off score of ≥ 5^11^ in the Tilburg Frailty Indicator questionnaire^12^ or b) need of language translator support during consultations.

**The control group** has access to usual care at the cardiac outpatient clinic: training (1 hour 2 times a week for 6 weeks), dietary training (2x3 hours), and cardiac education (2x3 hours). After or instead of hospital rehabilitation, the patient can be referred to municipal rehabilitation in Rehabilitation Center Albertslund: training (1 hour 2 times a week for 12 weeks), patient education (3x2 hours by a cardiac nurse and 1x2 hours by a dietician).

**Patients in the intervention group** will, in addition to usual care, have access to the abovementioned activities.

##### Registration of participation and sustainability

Partners register patients’ participation in all activities to monitor participation rates across sectors. Data from self-reported patient questionnaires are collected at baseline (background, and outcomes), 3, 6, 12, and 24 months (outcomes, and participation in rehabilitation activities). Physical activity in local community participation are also collected at 12 and 24 months after baseline.

### Questionnaires include:

- Tilburg Frailty Indicator^12^: physical, mental, and social vulnerability.
- Health Education Impact Questionnaire (HEIQ)^13^: positive and active engagement in life.
- SF-12^14^: Physical and mental health summaries (PCS and MCS).
- Nordic Physical Activity Questionnaire-short (NPAQ)^15^: physical activities in leisure time.

##### Qualitative data on patient experiences

The research team conducts qualitative semi-structured interviews with 20 patients to uncover their experience of the overall process, contact with health care professionals, the match between activities and rehabilitation needs, patient involvement, and intersectoral coordination. Patients will be selected based on criteria for maximum variation. Both patients with high and low levels of participation will be selected for interviews.

##### Qualitative data on implementation among professionals

Organizational characteristics and cultures as well as individual approaches among professionals will shape the implementation of activities. Field notes on adjustments in the intervention content, procedures, and changes in the context (e.g. organizational changes, changes in management/employees) will provide knowledge on the setting and the processes affecting the potential for the effect of the rehabilitation model. Observations and/or recordings of rehabilitation activities in hospital and municipality and Danish Heart Associations proactive counseling will provide insight into the actual content of the activities and the fidelity of the implementation. Focus groups with involved partners will uncover the professional’s experiences and reflections on screening and referral procedures, information exchange, intersectoral collaboration, and rehabilitation activities.

## 4. Evaluation

##### Descriptive analyses of the effect sizes

We will perform descriptive analyses of baseline characteristics. The primary outcome will be a) analyzed according to the intention-to-treat principle, b) sensitivity analyses of changes within/between the intervention and control groups will be carried out. If the power of data allows - explorative subgroup analyses will be carried out on those screened to be vulnerable. Estimated effect sizes will be calculated to inform future assessment of sample sizes in RCTs.

##### Qualitative evaluations

All data from workshops, interviews, and focus groups will be audio-recorded and transcribed verbatim. Interview transcriptions and field notes will be analyzed using systematic text condensation described by Malterud^16^. The analytical process will be inspired by principles from Collaborative Data Analysis^17^.

##### Patients’ experiences from the intervention

The analysis will assess whether patients experience their treatment and rehabilitation course as an integrated and coordinated effort helping them to live with their heart disease. The analysis will nuance the quantitative analysis and assess whether the model succeeds in tailoring rehabilitation activities to individual needs. The full rehabilitation package is not necessary or relevant for all patients with heart disease. Patients with vulnerabilities may need elaborate support and encouragement to deal with the mental and physical challenges of heart disease, while patients with resources may need less assistance. Rather they may need to be supported in continuing making healthy lifestyle choices and in using their local area and network to return to everyday life. In this analysis, gaps in the match between activities and patient needs will also be identified.

##### The implementation processes

The analyses of organizational experiences are inspired by the context, process, and outcome model ^18^. They will focus on the fit between the varying partnering institutions and the developed rehabilitation activities and collaboration procedures. The acceptability of the intervention among employees will be assessed continually to ensure their support for the implementation. Reach and fidelity of implementation will be assessed to provide relevant knowledge for interpreting and drawing conclusions on the potential of each intervention component.

##### Cost-effectiveness analyses

In order to compare the costs of the intervention towards the effects, a cost-effectiveness analyses will be carried out. This will be done by calculating a ratio where the denominator is the health gains measured by quality adjusted years of life (SF-12/SF-6D) and the numerator is the cost associated with the health gain obtained from the intervention, which will be the total cost of the intervention including resources used by the patients^19–21^.

## 5. Project organization - A partnership

HeRTA is anchored in the Intersectoral Prevention Laboratory (IPL) with expertise in supporting intersectoral partnerships and intervention development and execution.

HeRTA is a partnership between researchers at CCRP, the IPL, the cardiac outpatient clinic at Hvidovre Hospital, Rehabilitation Center Albertslund, and the Danish Heart Association. Local sports associations are part of the project at a collaborative level. The project receives sparring from an expert group and a patient advisory board.

**Researchers at CCRP** are Michaela Louise Schiøtz, Head of Section for Intersectoral Health Services Research; Hanne Birke, Ph.D., Postdoc with expertise in chronic disease (project manager); Karin Burns, a clinical coordinator with experience in supporting research in practice; Ida Foxvig, a research assistant with experience in partnerships and co-creation; Louise Meinertz Jakobsen, Ph.D., with expertise in interventions and rehabilitation.

**An expert group** provides professional feedback to the choice of research methods, outcome measurements, data collection, and analyses. Members are experts from Steno Diabetes Center Copenhagen, Hvidovre Hospital, Rigshospitalet, and the Immigrant Medical Clinic:

***Selina Kikkenborg Berg***, Professor of Cardiology at The Heart Centre, Rigshospitalet and University of Copenhagen. Main research areas: cardiac nursing, Psychocardiology, and Cardiac rehabilitation. Selina contributes with expertise in cardiac rehabilitation, patient needs and involvement, and complex health interventions.

***Ove Andersen***, Chief Physician, PhD, DMSci in internal medicine, infectious diseases and Clinical Professor at Copenhagen University. Head of the Clinical Research Center and Research Director at Amager-Hvidovre Hospital. Ove contributes with expertise in complex intervention studies, cross-sectional knowledge transmission, communication and patient involvement.

***Charlotte Demant Klinker***, Master of Science in Public Health, PhD, Team leader at Steno Health Promotion Research. Charlotte contributes with comprehensive experience in developing and evaluating complex, real-life interventions as well as expertise in partnerships and co-creation processes between organizations, professionals, and citizens.

***Hanne Winther Frederiksen***, Master of Health Sciences, Cand.Scient. San., PhD, Head nurse at the Immigrant Medical Clinic. Hanne contributes with expertise in migrant health and cardiac rehabilitation for non-Danish speaking citizens.

**A patient advisory board** with 12 members elucidates the patient perspective. The participating patients are selected so the differ on demographics and experiences with rehabilitation. The patient advisory board is involved throughout the project to ensure that decisions continuously focus on patient interests.

##

## 6. Ethics approval

A data handling plan has been accepted by the Knowledge Center for Data Reviews, the Capital Region of Denmark (journal-nr.: P-2020-905). Approval is not needed from the National Committee of Health Research Ethics ^22^. The study is registered at ClinicalTrials.gov^23^, identifier: NCT05104658.

## 7. Deliverables from the HeRTA feasibility study

The following deliverables are tangible outcomes of the project:

- Collaboration model across hospital, municipality, patient organization and local sports associations
- Feasible patient-targeted rehabilitation activities across sectors
- Identification of promising components to increase participation in rehabilitation and physical activity in local sports associations
- Tools to engage patients with vulnerability in rehabilitation

### Communication plan

We provide an evidence-based contribution to the debate on cardiac rehabilitation and social inequalities in health and healthcare. The target groups for our communication are the research community; health professionals across sectors including leaders and key persons; civil society (e.g. patient organizations); and cardiac patients.

Findings will be presented in meetings, conferences, and written material, such as fact sheets and articles in peer-reviewed scientific journals and popular science journals and channels. We will communicate through the Heart Foundation (Magazine and conference(s)) targeting cardiac patients and health professionals.

# Perspectives

Results from HeRTA will point to a model that is feasible and sustainable within the Danish healthcare system and forms a coherent rehabilitation pathway for people with cardiac disease. The model can be adjusted locally to fit the context in other rehabilitation locations and to ensure local stakeholder engagement. Our preliminary economic evaluation of financial cost and use of resources will operationalize the sustainability of the model. The results can be extrapolated to other chronic illnesses e.g. diabetes, COPD, and multimorbidity conditions. Chronic patients’ concerns and difficulties in terms of rehabilitation are mostly comparable across chronic illnesses.

HeRTA can form the basis for further targeted rehabilitation studies: a) rigorous RCT studies dissecting the effect of specific elements of the intervention that shows the greatest potential for positive benefits, and b) studies that test the generalizability across contexts to ensure transferability of results.

**References**

1. Broge, C. *Op imod en halv million hjertepatienter i 2020*. https://hjerteforeningen.dk/2014/04/op-imod-en-halv-million-hjertepatienter-i-2020/ (2014).

2. Schiøtz, M. L., Stockmarr, A., Høst, D., Glümer, C. & Frølich, A. Social disparities in the prevalence of multimorbidity - A register-based population study. *BMC Public Health* **17**, 1–11 (2017).

3. Kirolos, I. *et al.* Cardiac physiology in post myocardial infarction patients: the effect of cardiac rehabilitation programs—a systematic review and update meta-analysis. *Ann. Transl. Med.* **7**, 416–416 (2019).

4. Graversen CB, Eichhorst R, Ravn L, Christiansen SSR, Johansen MB, L. M. Social inequality and barriers to cardiac rehabilitation in the rehab-North register. *Scand. Cardiovasc. J* **51**, 316–322 (2017).

5. Rouleau, C. R. *et al.* A qualitative study exploring factors that influence enrollment in outpatient cardiac rehabilitation. *Disabil. Rehabil.* **40**, 469–478 (2018).

6. Frederiksen, H. W. Cardiac Rehabilitation Among Migrants. A Mixed-Methods Study. *phd thesis* 87 (2018).

7. Bellmann, B. *et al.* The Beneficial Effects of Cardiac Rehabilitation. *Cardiol. Ther.* 1–4 (2020) doi:10.1007/s40119-020-00164-9.

8. Meillier, L. K., Larsen, F. B., Nielsen, K. M. & Larsen, M. L. Socially differentiated cardiac rehabilitation: Can we improve referral, attendance and adherence among patients with first myocardial infarction? *Scand. J. Public Health* **40**, 286–293 (2012).

9. Murray, E. *et al.* NPT - a framework for developing evaluating and implementing complex interventions. *BMC Med.* (2010).

10. Damschroder, L. J. *et al.* Fostering implementation of health services research findings into practice: A consolidated framework for advancing implementation science. *Implement. Sci.* **4**, 1–15 (2009).

11. Gobbens, R. J. J., van Assen, M. A. L. M., Luijkx, K. G., Wijnen-Sponselee, M. T. & Schols, J. M. G. A. The tilburg frailty indicator: Psychometric properties. *J. Am. Med. Dir. Assoc.* **11**, 344–355 (2010).

12. Sutton, J. L. *et al.* Psychometric properties of multicomponent tools designed to assess frailty in older adults: A systematic review. *BMC Geriatr.* **16**, 55 (2016).

13. Osborne, R. H., Elsworth, G. R. & Whitfield, K. The Health Education Impact Questionnaire (heiQ): An outcomes and evaluation measure for patient education and self-management interventions for people with chronic conditions. *Patient Educ. Couns.* **66**, 192–201 (2007).

14. Ware, JE; Kosinski, M; Keller, S. *SF-12 : how to score the SF-12 physical and mental health summary scales*. (Lincoln, R.I. : QualityMetric Inc. ; Boston, Mass. : Health Assessment Lab, 2002).

15. Danquah, I. H., Petersen, C. B., Skov, S. S. & Tolstrup, J. S. Validation of the NPAQ-short - A brief questionnaire to monitor physical activity and compliance with the WHO recommendations. *BMC Public Health* **18**, 1–10 (2018).

16. Malterud, K. *Kvalitative metoder i medicinsk forskning: en innføring (3. edition). In Norwegian.* (Universitetsforlaget, 2011).

17. Cornish F, Gillespie A, Z. T. Collaborative Analysis of Qualitative Data. in *In The SAGE Handbook of Qualitative Data Analysis* (SAGE Public. Ltd., 2014).

18. Fridrich, A., Jenny, G. J. & Bauer, G. F. The Context, Process, and Outcome Evaluation Model for Organisational Health Interventions. *Biomed Res. Int.* 1–11 (2015).

19. Brazier, J. E. & Roberts, J. The estimation of a preference-based measure of health from the SF-12. *Med. Care* **42**, 851–859 (2004).

20. Maruish, M. *et al.* *User’s Manual for the SF-36v2 Health Survey*. (Quality Metric Inc., 2011).

21. Touray, M. M. L. Estimation of Quality-adjusted Life Years alongside clinical trials: the impact of ‘time-effects’ on trial results. *J. Pharm. Heal. Serv. Res.* **9**, 109–114 (2018).

22. The Danish National Committee on Health Research Ethics. The Danish National Committee on Health Research Ethics. https://en.nvk.dk/how-to-notify/what-to-notify (2019).

23. clinical trials. https://clinicaltrials.gov/ (2021).
